# Desmin expression in colorectal cancer stroma correlates with advanced stage disease and marks angiogenic microvessels

**DOI:** 10.1186/1559-0275-8-16

**Published:** 2011-12-05

**Authors:** Georgia Arentz, Tim Chataway, Timothy J Price, Zaipul Izwan, Gemma Hardi, Adrian G Cummins, Jennifer E Hardingham

**Affiliations:** 1 Department of Haematology-Oncology, The Queen Elizabeth Hospital, Woodville, SA 5011, Australia; 2 Department of Physiology, University of Adelaide, SA 5005, Australia; 3 Department of Physiology, Flinders University, Bedford Park, SA 5042, Australia; 4 Department of Medicine, University of Adelaide, SA 5005, Australia; 5 Department of Gastroenterology and Hepatology, The Queen Elizabeth Hospital, Woodville, SA 5001, Australia

**Keywords:** Colorectal cancer, 2D DIGE, desmin, biomarker, pericyte, angiogenesis

## Abstract

**Introduction:**

Biomarkers that improve stratification of colorectal cancer patients for adjuvant therapy versus resection alone, or that are predictive of response to therapeutic agents, have the potential to greatly improve patient selection for such therapies. The aim was to determine proteins differentially expressed within the malignant epithelial glands and closely associated stromal elements compared to matched normal mucosa, and to characterise the over-expression of one such protein as a potential biomarker.

**Methods:**

Protein from laser microdissected tumor and normal mucosa was analysed by two dimensional difference gel electrophoresis (2D DIGE) and mass spectrometry to determine differentially over expressed tumor proteins. Tumor over-expression of one such protein, desmin, was quantified using immunofluorescence staining in a larger cohort. Dual staining for desmin and vimentin, or desmin and von Willebrand factor, was performed to determine the cell type of interest.

**Results:**

Desmin expression was significantly increased between stage I and III tumors, (*P *< 0.0001), and stage II and III tumors, (*P *< 0.0001). Strong focal desmin expression was found in stroma directly adjacent to carcinomatous glands and microvessels. These cells showed co-localisation of desmin and vimentin in close association with cells expressing VWF, indicating they were pericytes. Significantly higher levels of desmin-positive pericytes were observed in late stage tumors, consistent with increased angiogenesis.

**Conclusion:**

Pericyte coverage of vasculature is a marker of vessel maturation, hence desmin expression may have use as a marker for microvessel maturation. Clinical trials will be needed to determine its use in identifying tumors that will be less responsive to anti-angiogenic therapy.

## Introduction

The staging of colorectal cancer (CRC) could be improved as up to 25% of patients deemed early stage (no regional lymph node or distant metastasis) relapse following presumed curative surgery [1]. This is likely caused by circulating tumor cells [2,3] or by established micro-metastatic disease in local lymph nodes or distant sites. Identification of a biomarker for more advanced disease in the primary tumor may result in down-staging the disease and thus identify a more appropriate selection of patients for increased surveillance and adjuvant therapy. Targeted therapy, such as anti-VEGF monoclonal antibody therapy, has shown a small but significant increase in progression-free and overall survival in a proportion of metastatic CRC patients in clinical trials [4], however many patients show resistance to anti-VEGF therapy. The identification of predictive biomarkers would therefore significantly aid in patient selection to improve efficacy and reduce the toxicity and cost of targeted therapy.

The analysis of alterations in the tumor tissue microenvironment has the potential to identify useful CRC biomarkers. Changes in the tumor microenvironment would influence the gene expression profile of surrounding epithelial and stromal cells [5]. Host factors and signalling between the tumor cells and neighbouring stromal cells play a role in angiogenesis, invasion and metastasis [5-7]. A change in the tumor microenvironment can lead to changes in the molecular cross-talk between epithelial and stromal cells (including endothelial cells), induced by heterotypic cell-to-cell contacts or signalling molecules by paracrine or autocrine actions (reviewed in [8]). Such changes in protein expression may provide useful biomarkers of tumor progression or as predictive biomarkers for tumor resistance to targeted therapy.

As tumor tissue is heterogeneous and may contain lymphoid aggregates and smooth muscle cells, it is important therefore to use laser micro-dissected (LMD) colorectal tissues for differentially expressed tumor marker identification [9,10]. We used laser microdissection (LMD) to collect areas of epithelium and closely associated stromal elements from tumor and matched normal mucosal tissue. Two dimensional difference gel electrophoresis (2D DIGE) was used to quantify the difference in the protein expression profiles of the samples to identify potential tumor biomarkers. This system encompasses a novel fluorescence 2D gel technique enabling multiplexing within the same gel, together with dedicated software for automated spot detection, background subtraction and quantitative spot volume calculations normalised to the internal reference sample. The software module matches images from different gels to provide statistical data on differential protein abundance. Multiplexing allows inclusion of a pooled reference sample, that is used for normalisation within the gel and comparisons between gels, a distinct advantage over conventional 2D electrophoresis.

The aims of this study were firstly to identify proteins differentially expressed in malignant epithelium and closely associated stromal elements, compared to matched normal mucosa using 2D DIGE and mass spectrometry (MS). Secondly, to analyse the over-expression of one tumor protein in a larger cohort of CRC samples as a means to validate the proteomic platform for differential protein identification, and thirdly, to characterise the cell type of origin.

## Materials and methods

### Patient specimens

Samples of tumor and matched normal mucosa (taken from the furthest resection margin) were collected from consecutive CRC patients undergoing resection surgery at The Queen Elizabeth Hospital, and snap frozen in liquid nitrogen. Additional tumor sections for immunostaining were obtained from archived formalin-fixed, paraffin-embedded tumor blocks. Patients that had received neoadjuvant therapy were excluded from the study. Ethics approval was received from the institutional Ethics of Human Research Committee (protocol 1993/59) and informed consent was obtained in all cases.

### Laser microdissection (LMD) and protein preparation for 2D DIGE

LMD was performed on paired tumor-normal tissues from 4 stage III cases. Frozen tissue embedded in OCT was cryo-sectioned (30 μm), placed on foil-framed PET membrane slides (Leica, Wetzlar, Germany), stored at -80°C, and thawed just prior to use. Sections for LMD were unfixed and unstained while adjacent sections were fixed and stained with haematoxylin for confirmation of morphology by a histopathologist. Using the Leica AS LMD system (Leica, Wetzlar, Germany), tissue was microdissected and collected in the cap of a 0.5 ml microfuge tube containing 40 μl of sample buffer (30 mM Tris, 7 M Urea, 2 M Thiourea, 4% (w/v) CHAPS). Areas of tumor which included neoplastic glands and adjacent stroma were selected for LMD, excluding extramural tumor extension to avoid capturing cells of the muscularis propria. After solubilisation overnight, samples were purified using a 2D Clean Up kit (Bio-Rad, Hercules, CA, USA), resuspended in an appropriate volume of sample buffer, and quantified using the EZQ Protein Quantification Kit (Invitrogen, Carlsbad, CA, USA).

### Scarce Labelling 2D DIGE

Five μg of each protein sample was diluted to 1 μg/μl in DIGE labelling buffer, pH 8.5, reduced with 1 μl of 2 mM TCEP (Sigma, St Louis, MO, USA), and labelled with 2 μl of 2 nmol Cy5 dye according to the manufacturer's protocol (GE Healthcare, Buckinghamshire, UK). Similarly, 5 μg of a pooled internal control (consisting of equal amounts of protein from all samples in the cohort) was labelled with Cy3, mixed with each of the individual tumor and normal samples and diluted in rehydration buffer (7 M Urea, 2 M Thiourea, 4% CHAPS, 1% IPG Buffer, 13 mM DTT) to a final volume of 450 μl. Samples were rehydrated overnight into 24 cm pH 3-7 non-linear IPG strips (GE Healthcare) at 50 V, followed by isoelectric focusing for approximately 70,000 Vhrs. Second dimension SDS PAGE was carried out at 350 V on 8-15% gradient polyacrylamide gels. Imaging of Cy3 and Cy5 labelled protein spots was performed using a Typhoon Imager 9400 (GE Healthcare). The matched tumor-normal gel images were cropped with ImageQuant v3 software (GE Healthcare) and loaded into the DeCyder™ v5 Batch Processor software. The software was set to calculate the average abundance change ratio of proteins across the 8 gels and the significance of the change using Student's paired t-test. The gels were analysed using the Biological Variation Analysis module of the DeCyder™ v5 software. For identification by tandem MS (MS/MS), protein spots were excised from a preparative gel containing 250 μg protein that had undergone 2DE as before. Proteins on the gel were visualised by a MS-compatible silver stain [11].

### Protein Identification

Protein spots excised from the preparative gel were washed 3 times with 25 mM ammonium bicarbonate/50% acetonitrile (ACN), dehydrated with ACN, and digested overnight at 37°C in 20 μl of 20 μg/ml trypsin in 25 mM ammonium bicarbonate/10% ACN. MS/MS was performed on a HTC ultra 3D Ion Trap (Bruker Daltonic GmbH, Bremen, Germany) fitted with a 0.075 × 150 mm C18 column (Zorbax 300 SB-C18, 3.5 μm, Agilent Technologies, Santa Clara, USA). Peak lists were generated using Data Analysis V 2.4 (Bruker Daltonik GmbH). The MSDB 20060831 database was searched using the MASCOT search engine, under the parameters of fixed carbamidomethylation of cysteines, variable oxidation of methionines, MS/MS and mass tolerance of 0.4 Da, and one missed trypsin cleavage.

### Immunofluorescence Staining

Verification of tumor over-expression of one protein (desmin) identified from the 2D DIGE study was performed using immunofluorescence (IF) on 36 tumor and matched normal mucosal tissues from 15 stage I, 11 stage II, and 10 stage III cases. Frozen tumor tissue was embedded in OCT and serial 7 μm cryo-sections were cut and placed on HistoGrip™ (Invitrogen, Carlsbad, CA, USA) coated slides. Western blotting was performed to assess the specificity of the anti-desmin mouse IgG1 monoclonal antibody (ab6322 Abcam, Cambridge, MA, USA) for use in IF. Fifty μg tumor protein was separated by 1 D PAGE, immunoblotted with the desmin antibody and detected using a fluorescent Cy3-conjugated secondary antibody (GE Healthcare). Sections for IF were fixed in ice cold acetone for 5 min, followed by washing in PBS. One section of each tumor was stained with Diff Quik (Invitrogen) for histological reference. Sections were blocked for the non-specific binding of both primary and secondary antibodies by incubation with Image-It™ Signal Enhancer (Invitrogen) for 30 minutes, followed by incubation with 10% goat serum (GS) in PBS for 30 minutes. Sections were washed thoroughly with PBS and treated with a 1:30 dilution of the anti-desmin mouse antibody in 10% GS/PBS at 4°C overnight in a humidity box. Bound antibody was detected with Alexa 488 conjugated anti-mouse (Invitrogen) diluted 1:500 in 10% GS/PBS for 1 hour at room temperature in a humidity box in the dark. As negative controls, sections were treated with 10% GS/PBS only or with a 1:20 dilution of an IgG1 isotype control antibody. All sections were counter stained with DAPI at 0.5 μg/ml (Sigma, St Louis, MO, USA) in the dark for 30 minutes, air dried, and mounted with ProLong^® ^Gold anti-fade reagent (Invitrogen).

Co-localisation of desmin and vimentin was assessed using a subset of 17 tumor tissues (8 stage I, 6 stage II and 3 stage III) chosen randomly from the cohort. Sections were treated as previously described with a 1:30 dilution of desmin antibody plus a 1:20 dilution of vimentin rabbit antibody (ab16700, Abcam). Bound antibodies were detected by Alexa 488 conjugated anti-mouse antibody (Invitrogen) diluted 1:500 and a Cy5 conjugated anti-rabbit antibody (GE Healthcare) diluted 1:200.

To determine desmin and VWF staining, 5 μm sections from formalin fixed paraffin embedded stage III tumors (n = 5) were placed on HistoGrip™ (Invitrogen) coated slides. Sections were washed twice in xylene, once in 50% xylene/50% ethanol, followed by washes in 100% ethanol, 95% ethanol, 70% ethanol, 50% ethanol, and deionised water. Slides were incubated at 37°C for 15 min in 0.05% trypsin/0.1% calcium chloride, pH 7.8 in a humidity chamber. Slides were thoroughly washed with deionised water, followed by PBS, blocked in 1% BSA/0.3% Tween-20 in PBS for 1 hour at room temperature in a humidity chamber, and incubated with a 1:30 dilution of desmin mouse antibody (ab6322, Abcam) and a 1:800 dilution of VWF rabbit antibody (ab6994, Abcam) at 4°C overnight in a humidity box. Slides were washed three times in PBS, incubated with anti-mouse-Alexa 488 diluted 1: 500 and anti-rabbit Alexa 568 (both from Invitrogen) diluted 1: 500 for 1 hour at room temperature in the dark. As negative controls, sections were treated with 1% BSA/0.3% Tween-20 in PBS only or with a 1: 20 dilution of IgG isotype control antibody. All sections were counter stained with DAPI at 0.5 μg/ml (Sigma) in the dark for 30 min, air dried, and mounted with ProLong^® ^Gold anti-fade reagent (Invitrogen).

### Immunofluorescence Analysis

Desmin staining was evaluated by imaging the entire area of each section at 10 × magnification in monochrome using an Olympus BX51 fluorescence microscope. Images were taken using 460-495 nm, 330-385 nm, 590 nm and 663 nm long pass filters to capture the Alexa 488, DAPI, Alexa 568, and Cy5 images respectively. Colour was added and images overlayed. Desmin staining was quantified using the AnalySIS LS Research phase analysis tool (Soft Imaging Systems, Olympus, Münster, Germany) which gave the area (μm^2^) and % area of the total image that was positive for desmin. This process was repeated to quantify the level of DAPI staining. Prior to phase analysis, the pixel threshold of each image was adjusted to only include areas of positive fluorescence, excluding background. The final percentage area positive for desmin staining was then calculated against the total cell area, as determined by the quantified level of DAPI staining. For each tumor the % area of desmin staining across the tissue section was averaged. As desmin is a smooth-muscle cell marker, areas of muscularis mucosa were excluded from analysis.

### Statistical Analysis

Student's paired t-test was used to assess differences in protein expression between tumor and normal LMD samples (DeCyder™ software, GE Healthcare) and to assess the difference in desmin expression among stage I, II and III tumors (Graph Pad Prism 5.0). A *P *value of < 0.05 was accepted as significant.

## Results

### 2D DIGE and protein identification

**Figure 1:**
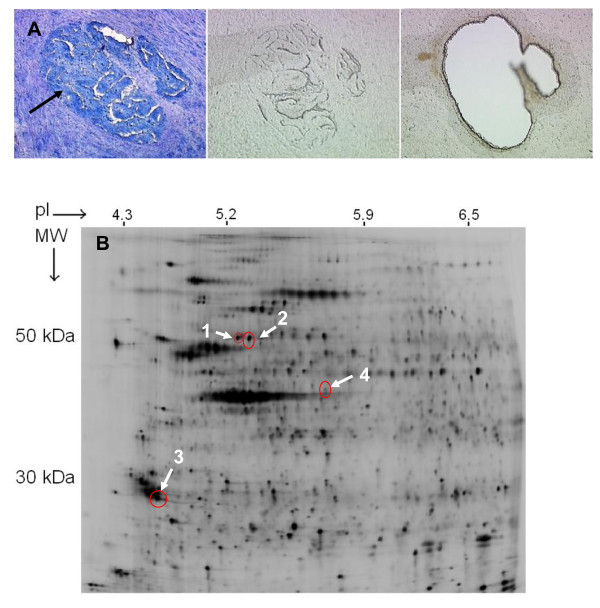
**Scarce labelling 2D DIGE on protein from laser microdissected tissue**. A, fixed and stained tumor tissue section (LHS), arrow points to malignant epithelial glands, unfixed and unstained section prior to LMD (centre), and after LMD (RHS). B, a scanned gel image of Cy5-labelled tumor protein with the circled proteins identified as significantly over-expressed in tumor versus matched normal mucosa across the 4 pairs (DeCyder™). Spot 1, identified by MS/MS as desmin shows an apparent molecular weight (MW) of 53 kDa, and isoelectric point (pI) of 5.21.

The average total protein yield of the tumor and normal samples following LMD was 41.5 μg (range 16.8-66.2) and 51.0 μg (range 19.4-82.6), from average areas of 28 mm^2 ^and 24 mm^2 ^respectively. An example of LMD is shown in Figure 1A. The 2D DIGE analysis showed 4 spots significantly higher in abundance across the 4 tumor samples (Figure 1B and Table 1). These proteins spots were identified by tandem MS (Table 1). Desmin (spot 1 Figure 1B) was identified with the highest Mowse score, the highest number of matched peptides and the largest sequence coverage and was selected for further analysis. The tumor-normal differential expression of this protein (*P *= 0.02) measured across the 8 gels is shown by graphical view (DeCyder™ software) in Additional file 1.

**Table 1:** Proteins higher in abundance in tumour relative to matched normal mucosa

		2D DIGE DeCyder™ calculations				Mass spectrometry		
**Spot number**	**Protein identified**	**Patient**	**T-N spot volume ratio**	**Ave. Ratio**	***P *value**	**Score**	**Peptides Matched**	**% seq. MS/MS**

1	Desmin	1	2.79	2.25	0.02	500 ^b^	11	31
	P17661^a^	2	1.19					
		3	1.58					
		4	3.45					

2	HSP60	1	3.51	2.57	0.01	12.4^ c^	7	10
	P10809	2	1.46					
		3	1.91					
		4	3.10					

3	14-3-3 β	1	1.48	2.0	0.01	234 ^b^	6	28
	P31946	2	1.57					
		3	1.65					
		4	3.46					

4	β Tubulin	1	1.59	2.98	0.017	117^ b^	2	5
	P07437	2	3.01					
		3	3.55					
		4	2.42					

a, UniProtKB database accession number; b, Probability-based Mowse score (Mascot search engine at Matrixscience.com); c, Protein Lynx score (Protein Lynx Global server) - an algorithm based measure of confidence of protein identity; d, Percentage sequence coverage for MS/MS identification.

### Quantification of desmin expression

**Figure 2:**
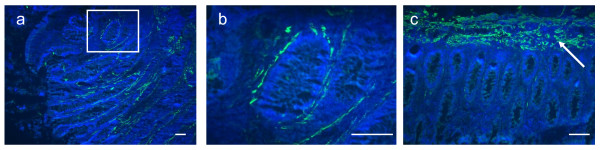
**Desmin immunofluorescence staining**. Representative examples of desmin staining with Alexa 488 (green) conjugated secondary antibody, and DAPI nuclei stain (blue) performed on colon tissues. A, tumor section showing stromal desmin staining. B, enlargement of white boxed area in A showing desmin positive cells surrounding a crypt. C, from the matched normal tissue section showing desmin-positive muscularis mucosa across the top of the section. Note the much reduced desmin expression in the normal epithelial tissue. Scale bar = 100 μm.

**Figure 3:**
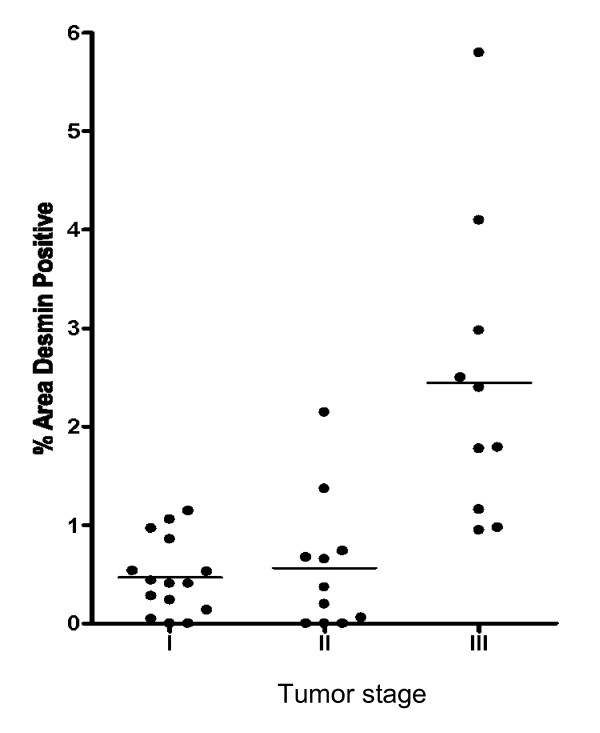
**Scatter plot of % area of desmin staining according to tumor stage**. Areas of muscularis mucosa and vasculature were excluded from analysis. Levels of staining were significantly increased between early stage (stage I and II) and stage III tumors, * P < 0.0001.

The origin and extent of the desmin expression was evaluated by immunofluorescence (IF) on tissue from stage I, II and III tumors. The desmin antibody showed a single band at the expected MW on Western blotting (Additional file 2). Desmin was expressed in the stromal cell area closely associated with the malignant epithelial glands of the tumor tissue (Figure 2). The desmin stained cells appeared in close association with malignant crypts. Low levels of stromal desmin staining were observed in the normal tissues, and this was generally sparse and discontinuous. The mean percentage areas of positive desmin staining (within the epithelial and stromal cell areas, excluding muscularis mucosa) for 15 stage I, 11 stage II and 9 stage III tumors are shown in Figure 3. Stromal desmin expression was significantly higher in stage III tumors when compared to both stage I and II tumors, *P *< 0.0001 (Student's t-test). There was no significant difference in the level of stromal desmin expression between stage I and II tumors (Student's t-test). While there was a significant correlation between the presence of a desmoplastic reaction and late tumor stage (*P *= 0.04, Fisher's exact test), no correlation was shown between desmoplastic reaction and high versus low level of desmin staining.

### Desmin, vimentin and VWF co-localisation studies

**Figure 4:**
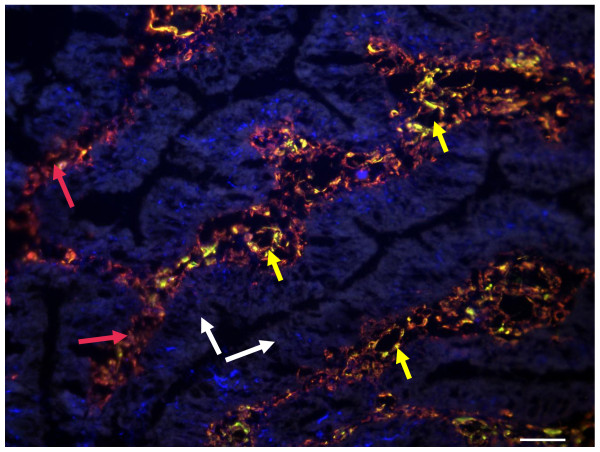
**Desmin and vimentin co-localisation**. Dual immunostaining staining performed on stage III tumor using Alexa 488 (green)- and Cy5 (red)- conjugated secondary antibodies for desmin and vimentin respectively, counterstained with DAPI (blue). Co-expression of desmin and vimentin by the same cell results in overlapping of the fluors, and appears yellow. Scale bar = 100 μm. Large amounts of vimentin staining appear between the epithelial crypts given vimentin is a fibroblast/mesenchymal marker and will stain stromal cells, endothelial cells and pericytes. The co-staining pattern of desmin and vimentin appears to be in cross and vertical sections of microvessels. Red arrows indicate areas of tissue stroma, white arrows malignant epithelial glands and yellow arrows cross-sectioned blood vessels showing co-localisation of desmin and vimentin.

**Figure 5:**
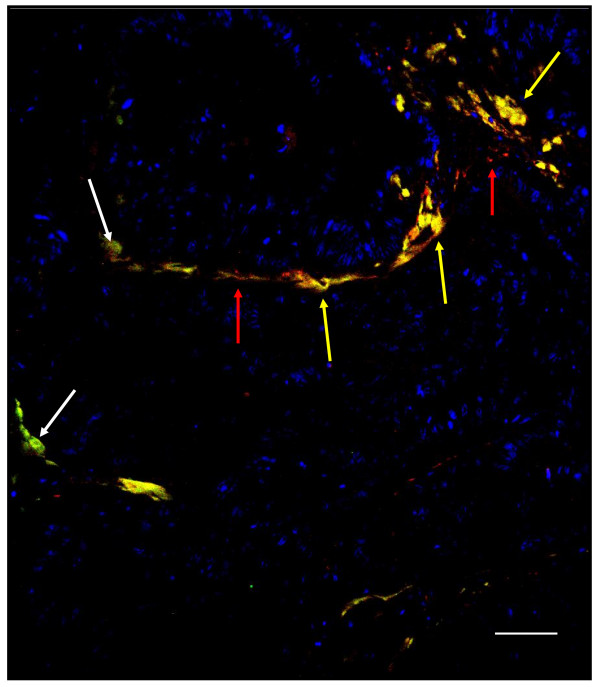
**Desmin and von Willebrand factor (VWF) immunostaining**. Dual immunostaining performed on stage III tumor tissue. Desmin was visualised with an Alexa 488 (green) conjugated secondary and VWF with an Alexa 568 (red) conjugated secondary, counterstained with DAPI (blue), scale bar = 100 μm. Co-localisation of the proteins appears yellow. Red arrows indicate cells positive for VWF, white arrows cells positive for desmin and yellow arrows co-localisation for desmin and VWF. The tumor section shows a blood vessel outlined by cells showing co-localisation of desmin (pericytes) and VWF (endothelial cells) in juxtaposition.

Desmin and vimentin double immunostaining was performed on 17 tumor samples to assess co-localisation. Desmin and vimentin co-localised in cells amongst the malignant crypts, in particular surrounding blood vessels (Figure 4). In addition there was strong vimentin staining of stromal cells amongst the malignant crypts as well as occasional stromal cells staining for desmin only. Desmin and VWF double staining commonly showed co-localisation to blood vessel walls in the tumor tissue (Figure 5) and in the normal mucosa from some stage III and IV tumors, but not from early stage tumors.

## Discussion

Proteomic determination of factors expressed by tumor cells and host stromal cells, either inherently or as a result of tumor-host interactions, has been shown to be useful in elucidating molecular pathways of tumor growth, invasion and metastasis (reviewed in [8]). In our 2D DIGE study, desmin was found over-expressed in colorectal tumors relative to matched normal mucosa. Previous reports of proteomic studies of differential expression of this protein in colorectal tumor tissues have shown conflicting results, from desmin over-expression [12], in agreement with our study, to reduced expression [13]. In other proteomic studies desmin was not identified among proteins over-expressed in colorectal tumors [14,15]. The varying results could be due to the fact that these studies were performed on tissue that had not been laser microdissected: the strong desmin expression by smooth muscle cells of the muscularis mucosae and muscularis propria would mask any differences between tumor and normal epithelium.

Desmin is a smooth-muscle type intermediate filament protein, expressed by smooth muscle cells, but also found expressed in fibrotic tissue in wound healing and in tumor 'desmoplastic' stroma, yet the origin of the cell type expressing desmin has been controversial [16]. Fibroblastic cells are the major component of tumor stroma and have been described variously as peritumoral fibroblasts, reactive (desmoplastic) stroma, cancer- or tumor-associated fibroblasts (CAF/TAF), and myofibroblasts. These cells belong to a functionally heterogeneous cell population and despite similar morphology, display distinct phenotypes in different pathological settings [16-19]. Myofibroblasts within the tumor stroma are thought to transdifferentiate from quiescent fibroblasts, driven by tumor-derived cytokines, in particular transforming growth factor (TGF)β [5]. Tumor stromal myofibroblasts have been shown to play a pivotal role in the switch from non-invasive to invasive cancer [16,18] and to promote and sustain tumor vasculature [20]. Using double-immunostaining we found different populations of cells within the tumor stroma i.e. vimentin-positive cells, as well as cells positive for both vimentin and desmin and some cells staining positive for desmin only (Figure 4). Previously, several myofibroblast subpopulations have been described based on their different expression of the intermediate filaments vimentin and desmin, with and without α-smooth muscle actin (α-SMA) [16,17,21]. These subpopulations have not been fully characterised but may reflect the continuum of differentiation from quiescent fibroblast (vimentin-positive, α-SMA negative, desmin negative) to myofibroblast (α-SMA positive, vimentin and/or desmin positive).

Desmin has also been described as a marker of pericytes found in association with blood vessels from the earliest stages of capillary sprouting and throughout angiogenesis [22,23]. Such cells have also been described as mural cells [24] or highly motile myofibroblast-like cells [25]. As a result of angiogenic signals, pericytes are recruited to developing endothelial tubes and express desmin in increasing amounts as they mature and elongate to form a stable sheath around the newly formed vessels [26,27]. The mature pericytes become focally embedded within the basement membrane adjacent to the endothelial cells [26,28] and are considered to be essential to angiogenesis both in normal physiology and in cancer [29,30].

The co-localisation pattern of desmin and vimentin co-staining surrounding microvessels in our study suggested the presence of pericytes (desmin-positive, vimentin-positive) tightly associated with the endothelial cells (also vimentin-positive) of microvessels (Figure 4). Double staining for desmin and the endothelial cell marker VWF [31] supports this conclusion (Figure 5). Pericytes and vascular smooth muscle cells (VSMC) comprise the mural cells that coat blood vessels, and it is now recognised that there is a continuum of phenotype from VSMC surrounding larger vessels to the typical pericytes coating capillaries and venules (reviewed in [29]). We therefore concluded that the desmin-positive, vimentin-positive cells were typical pericytes, rather than VSMC, coating the tumor micro-vessels. Taken together, our results show that the desmin expression is derived from both stromal myofibroblasts surrounding malignant crypts and from pericytes found in close contact with the tumor microvessels.

In our study there was a significantly higher level of desmin expression in stage III tumors when compared to both stage I and II tumors, suggesting a higher level of mature microvasculature in the late stage tumor tissue or a higher level of desmoplasia (or both). However there was no significant correlation between the level of desmin staining and the presence of desmoplasia in the tumors. This may be due to the fact that colon tumors with high desmoplastic grade have been shown to have a reduced number of microvessels (compared to tumors with low grade desmoplasia), but those vessels were mature with a higher level of pericyte coverage [32]. This would tend to balance out levels of desmin expression between tumors with high or low grade of desmoplasia, as we have found.

Mature pericyte-coated vessels are quiescent, and not dependent on angiogenic factors for survival [33,34], whereas uncoated vessels are considered to be unstable or immature, and responsive to angiogenic signals [35,36]. A significant proportion of metastatic CRC patients treated with anti-VEGF monoclonal antibody therapy or the tyrosine kinase inhibitors Sunitinib and Vatalanib, have shown poor response rates and generally only a modest effect on survival rates when used with standard chemotherapy [37]. Poor outcomes of these drug trials may be due to the presence of mature vasculature within the targeted tumors conferring a resistant phenotype [33,35,38-40].

In conclusion, desmin staining, identifying pericyte coverage and extent of mature tumor vasculature, may thus be worthy of further investigation as a biomarker to predict the efficacy of anti-angiogenic cancer therapy. Qualification of its use as a predictive biomarker will depend on a clinical trial incorporating desmin immunostaining of tumors from patients treated with or without anti-VEGF therapy.

## List of abbreviations

CRC: colorectal cancer; 2D DIGE: 2 dimensional difference gel electrophoresis; MS: mass spectrometry; LMD: laser microdissection; VWF: von Willebrand Factor; IF: immunofluorescence; CAN: acetonitrile.

## Competing interests

The authors declare that they have no competing interests.

## Authors' contributions

GA carried out the proteomic studies, IF staining and quantification, statistical analyses and participated in drafting the manuscript. TC contributed to the interpretation of proteomic studies and mass spectrometry and reviewed the manuscript. TP contributed to drafting the manuscript and critical review. ZI and GH performed IF staining. AC advised on the methodology and interpretation of IF quantification and critically reviewed the manuscript. JH conceived the study, contributed to its design and coordination, participated in drafting the manuscript and critical review. All authors read and approved the final manuscript.

## Supplementary Material

Additional file 1 **Graphical view of 2D DIGE protein spot abundance generated by DeCyder™ software**. The standardised log abundance of desmin is displayed on the Y-axis, with the normal (Group 1) and tumor (Group 2) samples displayed on the X-axis. The line represents the average increase in log abundance. Click here for file

Additional file 2 **Confirmation of the specificity and integrity of the desmin antibody by western blotting**. Fifty micrograms of whole tumour protein was separated by PAGE, immunoblotted with the desmin antibody ab6322 (Abcam) and detected using a fluorescent Cy3-conjugated secondary antibody. A single band slightly below 50 kDa was observed. The molecular weight markers were 'Dual Color' prestained SDS-PAGE standards (Bio-rad). Click here for file
