# Supplementary material for: Desmin expression in colorectal cancer stroma correlates with advanced stage disease and marks angiogenic microvessels
Source: Clin Proteomics. 2011 Dec 5;8(1):16. doi: 10.1186/1559-0275-8-16 (PMC3259060; doi:10.1186/1559-0275-8-16)
Supplement: Additional file 1 — Graphical view of 2D DIGE protein spot abundance generated by DeCyder™ software. The standardised log abundance of desmin is displayed on the Y-axis, with the normal (Group 1) and tumor (Group 2) samples displayed on the X-axis. The line represents the average increase in log abundance. [file 1559-0275-8-16-S1.DOC]

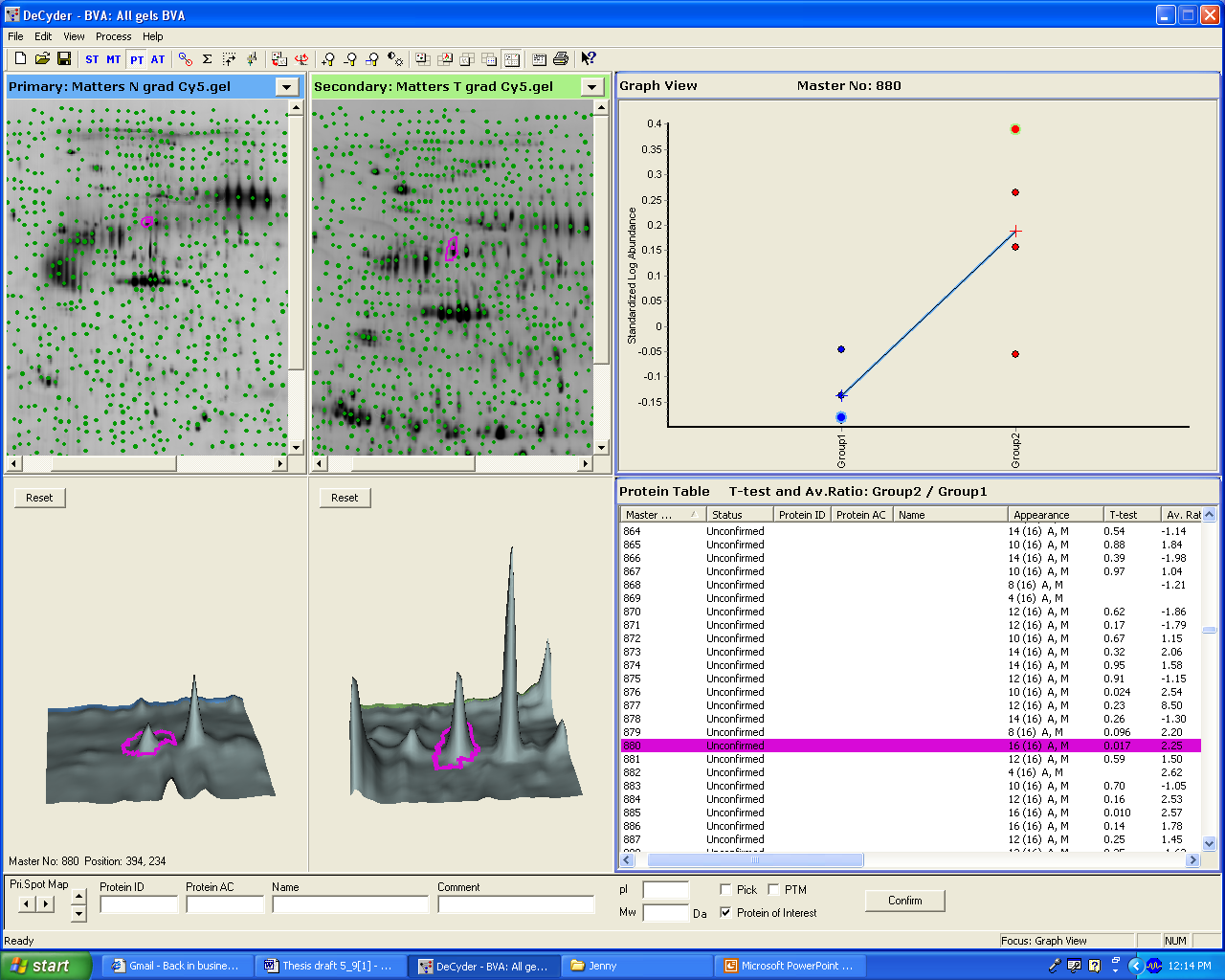


**Additional Files Fig. 1**

**Graphical view of 2D DIGE protein spot abundance generated by DeCyder™ software.** The standardised log abundance of desmin is displayed on the Y-axis, with the normal (Group 1) and tumor (Group 2) samples displayed on the X-axis. The line represents the average increase in log abundance.
