# Supplementary material for: Desmin expression in colorectal cancer stroma correlates with advanced stage disease and marks angiogenic microvessels
Source: Clin Proteomics. 2011 Dec 5;8(1):16. doi: 10.1186/1559-0275-8-16 (PMC3259060; doi:10.1186/1559-0275-8-16)
Supplement: Additional file 2 — Confirmation of the specificity and integrity of the desmin antibody by western blotting. Fifty micrograms of whole tumour protein was separated by PAGE, immunoblotted with the desmin antibody ab6322 (Abcam) and detected using a fluorescent Cy3-conjugated secondary antibody. A single band slightly below 50 kDa was observed. The molecular weight markers were 'Dual Color' prestained SDS-PAGE standards (Bio-rad). [file 1559-0275-8-16-S2.DOC]

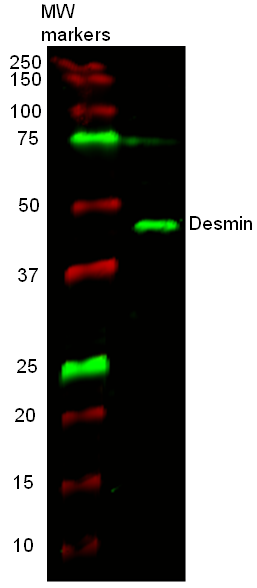


**Additional Files Fig. 2.**

**Confirmation of the specificity and integrity of the desmin antibody by western blotting.**

Fifty micrograms of whole tumour protein was separated by PAGE, immunoblotted with the desmin antibody ab6322 (Abcam) and detected using a fluorescent Cy3- conjugated secondary antibody. A single band slightly below 50 kDa was observed. The molecular weight markers were ‘Dual Color’ prestained SDS-PAGE standards (Bio-rad).
